# Computational lung modelling in respiratory medicine

**DOI:** 10.1098/rsif.2022.0062

**Published:** 2022-06-08

**Authors:** Sunder Neelakantan, Yi Xin, Donald P. Gaver, Maurizio Cereda, Rahim Rizi, Bradford J. Smith, Reza Avazmohammadi

**Affiliations:** ^1^ Department of Biomedical Engineering, Texas A&M University, College Station, TX, USA; ^2^ Department of Radiology, Perelman School of Medicine, University of Pennsylvania, Philadelphia, PA, USA; ^3^ Department of Biomedical Engineering, Tulane University, New Orleans, LA, USA; ^4^ Department of Anesthesiology and Critical Care, Perelman School of Medicine, University of Pennsylvania, Philadelphia, PA, USA; ^5^ Department of Bioengineering, University of Colorado Denver | Anschutz Medical Campus, Aurora, CO, USA; ^6^ Department of Pediatric Pulmonary and Sleep Medicine, School of Medicine, University of Colorado, Aurora, CO, USA; ^7^ J. Mike Walker '66 Department of Mechanical Engineering, Texas A&M University, College Station, TX, USA; ^8^ Department of Cardiovascular Sciences, Houston Methodist Academic Institute, Houston, TX, USA

**Keywords:** lung biomechanics, computational modelling, lung imaging, lung biophysical models

## Abstract

Computational modelling of the lungs is an active field of study that integrates computational advances with lung biophysics, biomechanics, physiology and medical imaging to promote individualized diagnosis, prognosis and therapy evaluation in lung diseases. The complex and hierarchical architecture of the lung offers a rich, but also challenging, research area demanding a cross-scale understanding of lung mechanics and advanced computational tools to effectively model lung biomechanics in both health and disease. Various approaches have been proposed to study different aspects of respiration, ranging from compartmental to discrete micromechanical and continuum representations of the lungs. This article reviews several developments in computational lung modelling and how they are integrated with preclinical and clinical data. We begin with a description of lung anatomy and how different tissue components across multiple length scales affect lung mechanics at the organ level. We then review common physiological and imaging data acquisition methods used to inform modelling efforts. Building on these reviews, we next present a selection of model-based paradigms that integrate data acquisitions with modelling to understand, simulate and predict lung dynamics in health and disease. Finally, we highlight possible future directions where computational modelling can improve our understanding of the structure–function relationship in the lung.

## 1. Introduction

Lung biomechanics is an active field of study that aims to understand the relationship between structure and function in the lung under normal and pathological conditions. Many pathological conditions—including acute respiratory distress syndrome (ARDS), emphysema and idiopathic pulmonary fibrosis—alter the structure of the lung acutely or in a delayed manner leading to lung functional decompensation [1]. The importance of understanding lung biomechanics is clear, especially in light of the COVID-19 pandemic. Before COVID-19, ARDS affected 200 000 individuals annually in the USA, with a mortality rate of approximately 40% [2]. The incidence of ARDS increased substantially with COVID-19, with 30% of COVID patients admitted to hospital having ARDS of which 75% were admitted to the intensive care unit [3]. ARDS mortality is strongly associated with ventilator-induced lung injury (VILI), potentially due to the interactions between micro- and macroscale elements of the lung [4]. Tools capable of describing and predicting the reciprocal relationships between lung structure and function are therefore critical to understanding the underlying mechanisms in disease progression, as well as for developing individualized therapies. The architecture of the lung is complex and fractal-like, with multiple length scales ranging from the liquid film containing surfactant on the surface of alveoli to large bronchi. The development of platforms that can integrate the mechanics of the lung at multiple scales and quantifiably relate lung function at the organ level to the responses of meso- and microstructural units is therefore essential to understanding the dynamics of the lung. Computational models [5–7] have emerged as powerful means to bridge multiple length scales and provide a detailed understanding of the lung structure–function relationship and its alterations in lung disease and injury.

**Figure 1.**
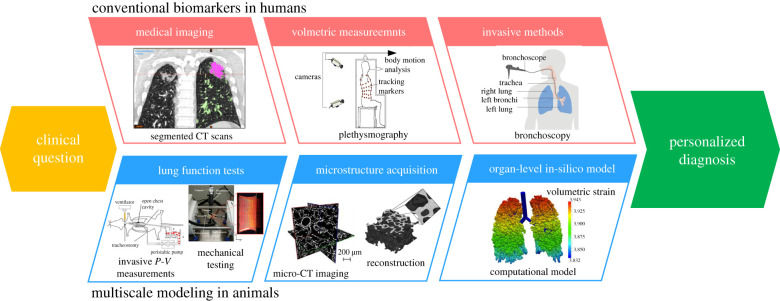
An idealized example of developing a computational model based on animal models and its application to patient diagnosis/treatment. CT, computed tomography; P-V, pressure–volume. Images taken from [6,8–12] and Cancer Research UK with permission.

Figure 1 shows the conceptual pipeline for using animal models and patient data to develop subject-specific computational lung models. The development of a comprehensive computational model that can describe lung biomechanics demands an understanding of the biophysical behaviour and microstructure of the surfactant system, the parenchymal tissue and the conducting airways, as well as how they relate to organ-level function. Preclinical lung injury studies offer valuable information regarding the multiscale structure–function relationship in the lung that would be infeasible to achieve in human subjects. These data, which include highly invasive measurements of structure and function, form the basis for computational models that provide insights into how minimally invasive clinical data can be used to deliver personalized lung injury prognosis and treatment.

This paper begins by reviewing the major topics relevant to the development of a physiologically faithful biomechanical computational model—including the anatomy of the lungs, mechanics of respiration and the process of data acquisition. The anatomy section provides insights into the development of material models of lung tissues that account for lung microstructure. The discussion of respiration mechanics assists with understanding the loading and boundary conditions required to simulate spontaneous respiration or mechanical ventilation. The data acquisition section reviews different modalities that can be used to estimate model parameters to capture physiologically accurate lung function. Following these sections, we review a selection of existing computational lung models, focusing on how different models combine data with existing knowledge on lung structure to generate and predict lung function in health and disease. We conclude our review by highlighting near-term goals in computational modelling of the lung that remain to be met. The scope of this review is focused on image-based and physiology-driven biomechanical models of lung function in health and disease; however, the broader landscape of respiratory modelling certainly includes other aspects of lung function, such as biochemical signalling and enzymatic processes, that remain to be covered in future reviews.

## 2. Anatomy of the respiratory system

**Figure 2.**
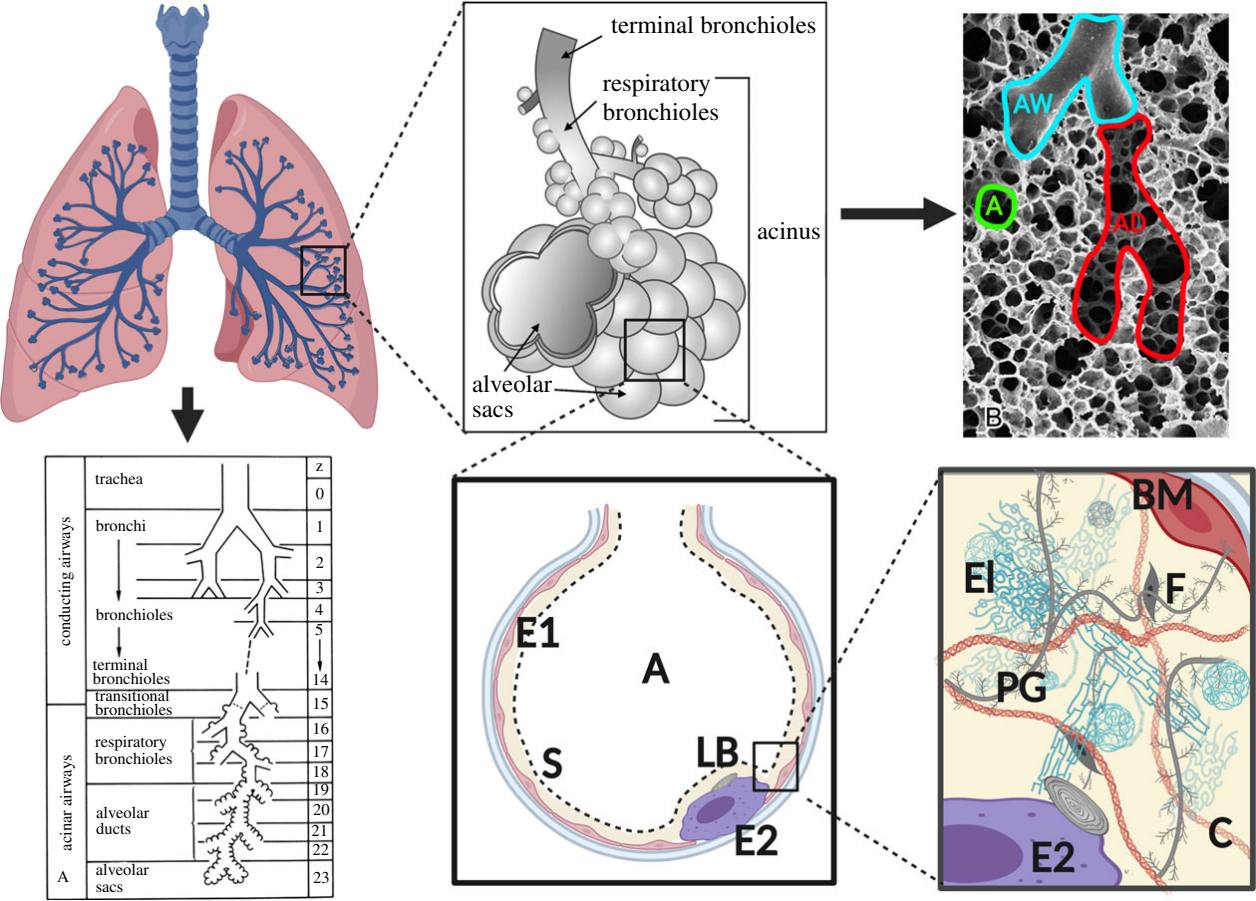
The hierarchical organization of the human lung components. The components are AW, airway; AD, alveolar duct; A, alveolus; E1, type I epithelial cell; E2, type II epithelial cell; S, air–liquid interface; LB, lamellar bodies. The components in the alveolar wall are El, elastin; PG, proteoglycans; C, collagen; F, fibroblasts; BM, basement membranes. Images taken from [13,14] with permission. Created using BioRender.

As a respiratory organ, the lung’s primary function is to transport oxygen from the atmosphere to the blood and remove carbon dioxide from the blood into the atmosphere. From a biomechanics perspective, the lung can be divided into two major components: the airways and the lung parenchyma. The conducting airways provide a pathway for air to travel from the atmosphere to the parenchyma, where the gas exchange between alveolar airspace and capillaries occurs. The effective biomechanical behaviour of the lung at both organ and parenchymal tissue scales strongly depends on the hierarchical structure of the lung (figure 2) and the biomechanical behaviour at the alveolar level. For this reason, we start by briefly reviewing several studies characterizing the architecture and material behaviour of the lung.

### 2.1. Conducting airways

The conducting airways comprise the nasal–oral cavity, the larynx, the trachea, bronchi and bronchioles. The general structure of the conducting airways is that of a branching tree beginning with the trachea, which subsequently divides into two bronchi. This pattern continues in a dichotomous pattern, where each airway divides into two ‘child’ branches. These branches are referred to as generations and have been numbered top-down or bottom-up [15,16]. The nature of branching and the number of generations is species-specific. The full airway tree in humans has an asymmetric branching pattern with 23 generations including the acinar airways (respiratory bronchioles). The conducting airways form the first 16 generations on average [17] with diameters starting around 14 mm for the main bronchus and going down to around 2 mm at the 15th generation [18]. The last seven generations comprise acinar airways that have alveolar entrances in their walls. In humans, the airways bifurcate into two nearly equal child airways with similar diameters, lengths and branching angles.

### 2.2. Lung parenchyma

The distal conducting airways connect to the acinar airways (figure 2), which are characterized by the presence of alveoli in the airway walls and lead to the alveolar ducts and surrounding alveoli. The alveolar ducts and alveoli that are fed by one terminal bronchiole are referred to as an acinus. Each acinar unit contains hundreds or thousands of alveoli, which are minute and delicate balloon-like structures perfused with a dense meshwork of capillaries. The alveolar structures provide a tremendous surface area and thin blood–gas barrier to facilitate efficient diffusive gas exchange. Human lungs contain an estimated average of 480 million alveoli [1], which translates to a surface area of the order of 100 m^2^. Collectively, the tissues that participate in diffusive gas exchange, namely the alveoli septa, are referred to as the parenchyma. To develop comprehensive biomechanical models of the parenchymal tissue, it is important to understand and isolate the mechanical contribution of each structural component in relation to the behaviour of the tissue as a whole.

Similar to most other soft biological tissues, the lung parenchyma exhibits a nonlinear stress–strain behaviour. A study by Zeng *et al.* [19] measured the stress–strain behaviour of lung parenchyma strips excised from humans, using an exponential-like constitutive relation to describe the behaviour. This nonlinear behaviour has been attributed to the realignment and straightening of load bearing fibres [20]. The authors also studied the creep and stress relaxation responses to measure parenchymal viscoelastic behaviour *ex vivo* [19]. They found that, in stress relaxation tests, tissue stress was reduced by 22–28% after 15 min. Interestingly, the study found regional variations in the lung tissue stress–strain behaviour as well.

The lung cells and extracellular matrix (ECM) form the alveolar walls. The ECM primarily consists of collagen, elastin and proteoglycans. A thin liquid film which covers a layer of epithelial cells lines the inner surface of the alveoli. This liquid film has a surface tension that is moderated by pulmonary surfactant and, along with the properties of the ECM, is an important determinant of the mechanical behaviour of the lungs. The properties of different components of the lung parenchyma were studied by Birzle *et al.* [9], who treated rodent parenchymal slices with collagenase and elastase to remove the collagen and elastin, respectively. The authors proposed that the lung parenchyma’s mechanical behaviour is determined by four components: collagen, elastin, ground substance (proteoglycans), and fibre interaction between collagen and elastin, and quantified the contribution of each component by treating the tissue with the appropriate enzyme. They found that elastin’s contribution was dominant at lower strains while collagen influenced the behaviour at both low and high strains. Neither the liquid film’s nor the surfactants’ effects were studied, as they were washed out during the treatment of parenchymal specimens. Below, we discuss the contributions of each ECM component and the liquid film to the mechanical behaviour of parenchymal tissues.

#### 2.2.1. Collagen

The collagen in the lung parenchyma consists primarily of type I and type III collagen. Collagen molecules assemble to form right-handed superhelix fibrils which are very stiff and have a diameter and length of 1.5 nm and 300 nm, respectively [21]. The mean collagen fibre diameter in human alveolar walls was reported to be 0.966 ± 0.481 μm [22]. These fibres show wavy organization at low lung volume. During inspiration, the collagen fibres are recruited via fibre re-orientation and straightening, exhibiting a strain-stiffening behaviour [20,23]. The variability in collagen fibre undulations and diameter leads to a wide range of time-constants in the viscoelastic behaviour of lung parenchyma [24]. Both quasi-static and dynamic collagen testing, in which tissue strips of the lung are treated with collagenase to isolate the contributions of collagen, has been reported [25–27]. Birzle *et al.* [25] found that the samples treated with collagenase had a larger drop in stiffness at higher strains, indicating a larger collagen contribution at this point, while elastin contribution was more significant at lower strains.

#### 2.2.2. Elastin

Elastin is another structural component of the parenchyma. Elastin self-assembles to form easily extensible cross-linked fibres, although its three-dimensional (3D) geometry has not been well documented [28]. The mean diameter of elastin fibres in human alveolar walls was reported to be 0.973 ± 0.472 μm [22], and the statistical distribution of diameter values within the parenchyma was found to be skewed towards thinner fibres with a long tail, similar to that of collagen fibres [22], although the elastic modulus of elastin is approximately two orders of magnitude smaller. Unlike the curly collagen fibres, elastin remains linearly elastic up to 200% strain [22], with values reported to be around 1 MPa. The relatively low elastic modulus of elastin is believed to be due to its amorphous structure [22]. Furthermore, because of this linear elastic behaviour, elastin substantially contributes to lung elastic recoil at lower inflation levels. Elastin is connected to collagen fibres via microfibrils or proteoglycans to form an interconnected network in the septa.

#### 2.2.3. Proteoglycans

The proteoglycans form an amorphous matrix [29] in which collagen and elastin fibres are embedded and appear as opacities in transmission electron microscopy (TEM) imaging. They are known to play several important biological roles: they influence intracellular signalling by acting as receptors on the surface of epithelial cells that facilitates cell–cell adhesion and cytoskeleton organization [30]; they can also bind to growth factors and proteins to regulate the secretion of proteins involved in tissue remodelling. While the interactions between proteoglycans and collagen determine the growth of collagen in the lateral and axial directions [31,32], the nature and extent of proteoglycans’ role in lung biomechanics remain to be fully understood. However, existing studies suggest that their direct contribution to lung parenchyma stiffness is not significant compared with that of collagen and elastin, although they play a significant role in stabilizing alveoli (mechanics, structure and function) [33].

#### 2.2.4. Liquid film

The parenchyma is lined with a thin liquid film that contains pulmonary surfactant produced by type II alveolar epithelial cells. This liquid film is known to substantially contribute to lung elasticity (stiffness), with several studies reporting that recoil pressure decreases significantly in saline-filled lungs when compared with air inflation, due to the lack of surface tension forces from the liquid film in saline-filled lungs [34,35]. The surfactant biophysics plays a key role in respiration by regulating the surface tension of the liquid lining layer. The surface tension varies during normal breathing by the amount of surfactant released by the epithelial cells, the molecular-scale structure of the surfactant in the alveolar space and the time-course variation of alveolus surface area. At low lung volumes, the surface tension is lowered through dynamic compression of the surfactant monolayer. Since the surface tension is inversely proportional to the surface concentration of surfactant, dynamic compression reduces surface tension to near zero, which is essential for preventing alveolar collapse (derecruitment) [36]. In general, the liquid film has been modelled as acting in parallel to the elastic behaviour of the parenchyma, in opposition to inflation [7,34]. Many lung models have incorporated the effect of varying surface tension [7,37–41] to mimic the observed hysteresis in the lung pressure–volume relationship. However, this remains a challenging area for computation due to the tremendous disparity in length scales, thin-film interfacial flows, surfactant dynamics and fluid–structure interactions in the lung.

### 2.3. Pleural membranes

Both lungs are covered in two serous membranes separated by pleural fluid. The outer membrane is referred to as the parietal pleura and is attached to the intercostal muscles and diaphragm. The inner membrane is called the visceral pleura and surrounds the lung parenchyma. Both membranes contain mesothelial cells which secrete the pleural fluid enabling the lungs to slide during expansion within the pleural cavity. The visceral pleura is also composed of elastin and collagen and hence contributes to the mechanical behaviour of the lungs during respiration. A study by Stamenovic [42] reported that the serous membranes could carry around 30% portion of the shear loading. The report additionally suggested that the membranes’ contribution might be higher for other types of loading. Another study by Melo *et al.* [43] used atomic force microscopy (AFM) and found that the visceral pleura has a significantly higher stiffness than that of alveolar walls represented by the ranges of 56.6 ± 4.6 to 99.9 ± 11.7 kPa and 27.2 ± 1.64 to 64.8 ± 7.1 kPa, for the membrane and the walls, respectively.

## 3. Mechanics of respiration

Understanding the mechanics of spontaneous breathing is essential to correctly account for the driving forces and identify the precise boundary conditions when modelling respiration. This section will also briefly discuss the differences between mechanical ventilation and spontaneous breathing. Simulating lung function during ventilation is important when studying/modelling conditions such as ARDS [44], in which the pressure and flow values supplied by the ventilator can further damage the lungs through VILI, leading to worse outcomes.

Before delving into the details of respiration, it is important to briefly list the different volume and pressure parameters commonly measured to assess organ level lung function [45]. Normal breathing in a subject at rest is commonly referred to as tidal breathing, and the volume of air moved in and out of the lungs is referred to as the tidal volume (*V*_*t*_). The volume of air present in the lungs at the end of expiration during tidal breathing is called functional residual capacity (FRC). Total lung capacity (TLC) is the total volume of air that the lungs contain after maximal inspiration, while residual volume (RV) is the volume of air remaining in the lungs at the end of a maximal forced expiration. The difference in volume between RV and TLC defines the vital capacity. These common clinical measures of lung health are typically measured using plethysmography.

**Figure 3.**
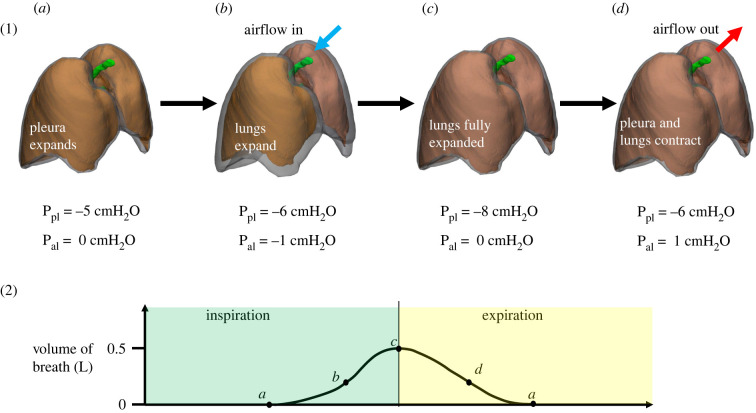
The change in alveolar and pleural pressure during respiration and how it affects air flowing in and out of the lungs. al, alveolar; pl, pleural. Images adapted from [46].

Spontaneous respiration is driven by the behaviour of the tissue surrounding the lungs. The lungs are also surrounded by a thin layer of fluid in the pleural space and thus are subjected to pleural pressure on their outer surface. During inspiration, the inspiratory muscles (consisting of the diaphragm and intercostal muscles) contract, causing the pleural space to expand [5]. The expansion of the pleural space causes the pleural pressure to fall, leading to the expansion of the lungs and a drop in alveolar pressure below atmospheric pressure, which in turn causes airflow into the lungs. During expiration, the diaphragm and intercostal muscles relax, causing an increase in pleural pressure which in turn increases alveolar pressure and drives air out of the lungs. During forced expiration, muscle contraction can provide additional positive pressure to the pleural space, further increasing both alveolar pressure and the rate of expiration. Changes in alveolar and pleural pressures during a respiratory cycle are illustrated in figure 3.

Changes in transpulmonary pressure (alveolar pressure minus pleural pressure) are a driving force of respiration and should therefore be incorporated into computational models to properly simulate *in vivo* lung physiology. Changes in transpulmonary pressure throughout the respiration cycle translate to lung motion which is measurable through medical imaging. For example, displacement measurements on costal and diaphragmatic surfaces at end-inspiration have been used to estimate changes in alveolar pressure [5,47]. Using a similar method based on computed tomogrpahy (CT) scans, Tawhai *et al.* [48] prescribed approximate normal displacements on the parietal pleural surface at FRC and TLC in their lung model to simulate spontaneous respiration. In contrast to prescribing the displacements directly on the lungs, prescribing the displacement on the parietal pleural membrane, which is separated from the lungs by pleural fluid, allows for lateral movement of the lung during respiration. In summary, spontaneous respiration models use either lung displacement or pleural pressure as the input to predict airflow into the lungs and changes in alveolar pressure. However, it should be noted that pleural pressure, and hence transpulmonary pressure, is not uniform, but varies in the vertical direction across lung height [49–51]. Computational models must therefore account for this vertical gradient to accurately simulate lung motion if pleural pressure is used as the input.

Mechanical ventilation is used when spontaneous breathing is insufficient to maintain gas exchange. Unlike in a free-breathing subject, where the contraction of the surrounding muscles drives inhalation, mechanical ventilation is driven by directly pumping air into the trachea at a controlled pressure and/or volume. The resulting inflation of the lungs is opposed by the resistive and elastic properties of the lungs, the surfactant in the alveoli and the viscoelasticity of the thorax. Although the overall concept is simple, ventilation parameters must be carefully selected for each patient to reduce the risk of VILI. Positive end expiratory pressure (PEEP) is a common biomarker in mechanical ventilation, defined as the pressure in the lungs above atmosphere at end-expiration. PEEP is one of the most important parameters set during mechanical ventilation with improper values of PEEP leading to VILI [52]; in particular, excessive PEEP may lead to overdistension (volutrauma), while low PEEP may lead to the cyclic collapse and reopening of alveoli and small airways (atelectrauma). Computational models of ventilation [52,53] use time-dependent flux (flow) or pressure boundary conditions at the trachea to simulate mechanical ventilation to optimize PEEP values in a patient-specific manner.

Assimilation of patient-specific physiological and imaging data into computational models enables multiscale quantification of the structure–function relationship in the lung and the advancement of *in silico* lung models for use in precision pulmonary medicine. Available methods for obtaining patient-specific data relevant to lung computational models are presented in the following section.

## 4. Data acquisition

**Figure 4.**
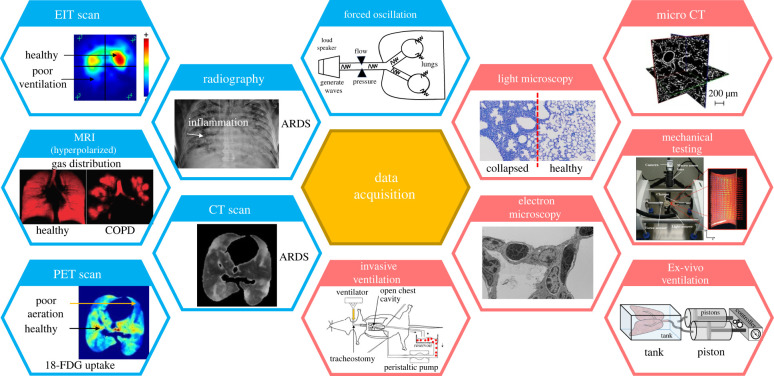
Various data acquisition methods used in clinical and preclinical settings. Blue and red headers are used to separate non-invasive from invasive methods. MRI, magnetic resonance imaging; EIT, electrical impedance tomography; PET, positron emission tomography; COPD, chronic pulmonary obstruction. 18-FDG, [^18^F]-fluoro-2-deoxy-d-glucose; note that there is lower uptake of 18-FDG and lower radioactivity in regions with poor aeration in the PET scan. Images are taken from [8,9,12,54–60] with permission.

Computational lung models depend on multi-modality datasets, potentially collected at different length scales, to reliably simulate and predict the structure–function relationship in the lung. These datasets provide essential population-level and subject-specific information on components of the lung from the nasal cavity to the alveoli. For this reason, various invasive and non-invasive data collection methods (figure 4), from pressure–volume (P-V) measurements to imaging, have been used to build and calibrate models and estimate the involved parameters. Below, we review a variety of data that can be collected from lung mechanical function measurements and lung imaging and discuss how such data can be incorporated into subject-specific lung models. *Ex vivo* biomechanical testing and imaging of harvested parenchymal and airway tissues are additional invasive techniques, commonly used in animal studies, that can provide important modelling data [33,61,62]. Such studies have been previously described [63] and will not be extensively discussed here.

### 4.1. Lung mechanical function

Accurate measurements of the lung volume are crucial to assess lung function in health and disease. Not only the spontaneous lung volume but also the time-course variation of the lung volume within several respiratory cycles plays an important role in assessing lung mechanics due to the viscoelasticity of the parenchymal tissue. Plethysmography and spirometry are common methods to non-invasively measure lung volumes—and resistances, in the case of plethysmography [63]. Both of these tests are also broadly applicable, as they are typically performed on conscious, free-breathing subjects. In plethysmography, the patient is enclosed in a sealed box where they breathe into a mouthpiece. Plethysmography estimates important volumetric lung capacities, as well as airway resistance, using pressure measurements in the box and Boyle’s Law. Spirometry is an alternative method for performing volumetric measurements without the use of an enclosing box but does not approach plethysmography’s level of detail.

The forced oscillation technique (FOT) [64,65] is another common modality for measuring lung mechanical function *in vivo* in humans and animals, or *ex vivo* in harvested lungs. Pressure oscillations are produced by a pump or speaker and are applied to the lungs through a mouthpiece or tracheostomy tube. The pressure input can be a single sinusoidal wave or a set of waves with multiple frequencies of interest. The pressure is then measured at the opening of the mouthpiece. Temporal pressure and flow measurements are transformed to the frequency domain using Fourier analysis and used to calculate the mechanical impedance (Z). The real part of this impedance describes the resistance, while the imaginary component describes the reactance, or elastance, at the frequencies applied to the respiratory system. In small animal models, the constant phase model is fitted to the multi-frequency impedance measurements [66] to compute Newtonian flow resistance, tissue elastance, tissue damping and gas inertance. A distinct feature of the FOT is that it can be applied to spontaneously breathing patients without confounding the results as long as the applied oscillations fall outside the frequencies of the underlying respiration.

The gold standard for measuring respiratory mechanics is invasive mechanical ventilation of a chemically paralysed subject. The paralytic is applied to prevent spontaneous breathing efforts that mask the true resistive and elastic properties of the respiratory system. Measurements conducted during ventilation can include simple stepwise quasi-static P-V measurements that describe the quasi-static compliance of the respiratory system (*C*_st_), defined as the change in volume per change in pressure at zero flow. Dynamic measurements, such as single-frequency sine waves, are commonly fitted to the single compartment lung model to evaluate respiratory system resistance (*R*_rs_), which includes both tissue and airway resistance as well as the respiratory system elastance (*E*_rs_), the latter of which is defined as the change in pressure per change in volume (the inverse of compliance). In animal models, the FOT is also frequently used to probe respiratory system mechanics during ventilation. However, this technique has yet to gain broad application in mechanically ventilated human subjects.

Similar volumetric measurements can be performed on harvested whole lungs enclosed in a box that are inflated and deflated using a pump or syringe. By excising the lungs from the thoracic cavity, it is possible to evaluate the properties of the lungs independent of the contributions of the chest wall. This measurement provides the P-V relationship from which various lung properties can be calculated as described above for ventilated subjects, including lung compliance (*C*_*L*_) and lung resistance (*R*_*L*_) [23,67]. Transpulmonary pressure is similarly easy to determine, and the bulk modulus (*B*) can be calculated from the P-V curve as well. Recently, Sattari *et al.* [68] applied this approach to measure the organ-level viscoelastic behaviour of harvested lungs. Sealing the lungs in a tank, with one piston connected to the trachea and one piston connected to the tank, the authors created a device that could simulate spontaneous breathing and mechanical ventilation based on the piston chosen to provide the volume-controlled air flow. The driven piston was then used to measure the changes in pressure and volume caused by the expansion of the lungs. The authors investigated different deformation fields induced by the two types of ventilation in both murine and porcine lungs [57,68] using a speckle pattern on the costal surface along with digital image correlation. These studies offer the possibility of identifying and using appropriate kinematic metrics to optimize mechanical ventilation protocols in human lungs.

### 4.2. Imaging techniques to characterize lung structure and function

Chest X-ray (CXR) and thoracic CT scans are the most commonly used non-invasive imaging modalities for studying lung structure. Other imaging modalities used for both clinical and translational studies of the lung include magnetic resonance imaging (MRI), positron emission tomography (PET), electric impedance tomography (EIT) and lung ultrasound (LUS). While CXR’s advantages include its speed and ease of use, its diagnostic ability to differentiate between lung conditions such as ARDS, pneumonia, atelectasis and pleural effusion is poor [69]. However, CXR is still widely used in lung imaging to detect secondary complications such as pneumothorax and displacement of devices [70]. Thoracic CT scans use X-ray beams at different angles that are then reconstructed to create a 3D image, offering both higher resolution and the ability to obtain quantitative imaging parameters. More specifically, thoracic CT scans assign a numerical value to each volume element (voxel) that describes X-ray attenuation, enabling the quantitative estimation of lung aeration. CT scans are used clinically in the diagnosis of chronic obstructive pulmonary disease (COPD) [71–73], interstitial lung disease [60,74], emphysema [75–77], asthma [78] and ARDS [79–81].

Lung deformation has a complex and significantly heterogeneous pattern due to the lung’s spatially heterogeneous mechanical properties, unequal time constants in different lung regions and the non-uniform expansion of the thoracic cavity during respiration. Thoracic CT scans can be used to quantify this deformation by imaging the lungs at certain capacities (e.g. end-inspiration and end-expiration) to produce lung geometries at different phases in a respiration cycle [5,47,48]. The displacement field of the lung parenchyma can be calculated from this deformation, and image registration techniques can subsequently be used to create a time-dependent 3D displacement field which can serve as an important boundary condition in simulating spontaneous or ventilated breathing [82].

MRI can produce images with similar resolution to CT, but without ionizing radiation. Conventional MRI produces contrast by detecting water-bound protons in tissue, which depend on tissue proton density. However, lung tissues have low proton density and also yield high susceptibility to artefacts from the gas–tissue interface, resulting in inadequate image contrast and incomplete morphological information. However, recent advances have enabled the use of noble gases, including He-3 and Xe-129, as contrast agents, improving our ability to image alveolar spaces [83]. Oxygen-enhanced MRI has also been developed to visualize ventilation. Similar to oxygen, these gases travel through alveolar spaces, septal tissue and blood, allowing the calculation of parameters such as septal wall thickness and alveolar surface area. Hyperpolarized lung functional MRI allows for the evaluation of regional ventilation via the estimation of gas distribution in the patient’s lung, and MRI scans in patients with diseases such as asthma, COPD, and cystic fibrosis clearly show that not all areas of the diseased lung are equally recruited. A study by Washko *et al.* [84] showed comparisons between (hyperpolarized) MRI and multi-detector CT (MDCT) scans for a patient, reporting that MRI was able to detect the lack of function in certain lobes, while CT was not. MRI can thus be used in place of CT in certain situations to minimize patients’ exposure to radiation while obtaining additional functional information such as gas distribution.

EIT is another important bedside imaging technique that can provide semi-continuous information about the regional changes in lung resistivity based on differences in ventilation from the reference state. EIT can display poorly ventilated regions of the lungs, identified by a lack of change in impedance, as well as alveolar overdistension, identified by a large change in impedance. A study by Roth *et al.* [52] used EIT as a validation tool for their computational model and found that the measured change in impedance was correlated to ventilation and, by extension, to alveolar strain. This study suggested that EIT could be used for continuous bedside monitoring of regional lung activity to prevent alveolar overdistension (i.e. volutrauma).

In addition to *in vivo* medical imaging methods, microscopic imaging such as light microscopy, electron microscopy and micro-CT are commonly used *ex vivo* in preclinical studies to image harvested tissues and analyse lung microstructure. Micro-CT [11,59,85,86] imaging has been used in combination with computational modelling to image and reconstruct porous parenchymal tissue to study the relationship between macroscopic and microscopic strains. Light microscopy (LM) can be used to view the alveolar mechanics dynamic *in vivo* [87] or uses excised tissue that is sectioned and mounted on glass slides. Because of its relative simplicity and low cost, LM is the most commonly used technique in preclinical studies. However, LM has also been used clinically on stained biopsied tissue to assess changes to lung microstructure in diseases like ARDS [88–90]. TEM is a higher-resolution imaging technique that can be used to study the sub-cellular structures in the lung parenchyma [91–93]. The primary limitation of conventional EM is its lack of 3D information due to the minimal thickness of the slices used (60–90 nm). However, recent developments in ‘volume electron microscopy’ have enabled 3D reconstruction and visualization of septal wall morphology [94,95].

### 4.3. Mechanical testing of the parenchyma at tissue and alveolar wall levels

In addition to its geometry and organ-level function, the lung’s material properties are important when developing discretized computational models of lung biomechanics. These material properties include stiffness, density, viscoelasticity and porosity, and can be obtained from experimental testing of excised tissue. Several studies have conducted mechanical testing of parenchymal tissue (e.g. [96–99]); however, given the scope of this review, we will focus on studies that used *ex vivo* data to develop material models for parenchymal tissues. Birzle *et al.* [9,25,100,101] measured the viscoelastic mechanical properties of lung parenchyma in uniaxial tension and developed a structurally based material model, differentiating between the contributions of different components of the parenchyma (including collagen and elastin fibres) to tissue-level behaviour. The mechanical testing of the parenchyma at the tissue level is complemented by its mechanical characterization at the alveolar wall level, which together could provide an understanding of force transition in the parenchyma across length scales once combined with modelling. The alveolar wall-level tests could also provide further insights into the contributions of collagen and elastin fibres to the mechanical behaviour of the parenchyma. An example of such tests was reported by Jorba *et al.* [102] who used AFM to investigate the properties of alveolar walls prepared from rodent parenchymal tissues. Interestingly, they found that the septal wall stiffness was an order of magnitude larger than that of the parenchymal tissue and that the stiffness was dependant on lung strains. The information about the behaviour of a single alveolar wall is valuable in the development of image-based, microscale biomechanical models of the parenchyma discussed in the next section. Lastly, due to differences in structure, the biomechanical properties of the airways diverge from those of the parenchyma. Eskandari *et al*. [103,104] investigated the mechanical properties of porcine airways via biaxial stretching of excised airways, developed a material model that takes into account the contributions of both the matrix and the fibres in the airways, and found that the airways exhibited nonlinear anisotropic and heterogeneous behaviour. The incompressible material model resulted in good agreement between model estimations and the experimental data.

## 5. Computational modelling

Computational modelling offers a unique platform for integrating imaging, respiratory mechanics and structure–function data measured at different length scales to understand, simulate and predict lung dynamics in both health and disease. Roughly speaking, there are two main approaches to computational lung modelling: compartmental (reduced-order) and anatomically based (high-fidelity). Compartmental models often focus on establishing relatively simple mathematical relationships between lung properties, such as relating organ-level pressure and volume measurements through lung resistance and elastance. They also offer a suitable platform for studying the multiphysics response of the lung, e.g. by integrating lung mechanics, pulmonary circulation and gas exchange. These models hold significant translational potential for lung diseases such as ARDS, where model prediction must be achieved in a timely manner and with limited data. Anatomically based computational modelling of the lung typically involves reconstructing the lung anatomy from medical imaging, acquiring subject-specific microstructure from *ex vivo* experiments, and applying appropriate boundary conditions obtained from imaging and invasive lung function assessments. This modelling paradigm enables a detailed simulation of the multiscale and high-fidelity mechanics of different lung components, capturing the translation of physiological or pathophysiological mechanisms from the microscale (the alveoli and terminal bronchioles) to organ-level behaviour. In contrast to the zero-dimensional (0D) models, well suited for clinical applications, image-based high-fidelity models may be more appropriate for preclinical mechanistic studies in which there is ample time and data for model parametrization. Combining the feasibility of 0D models with the subject-specificity of image-based models, therefore, remains to be an important unmet milestone. In this section, we briefly review reduced-order, or 0D, models, addressing the multiphysics nature of lung function. We then cover multiscale computational models, starting with discrete micromechanical models of alveolar tissue, followed by continuum-level, or ‘homogenized’, models of the lung. Finally, we discuss select models that focus on flow in the airway tree.

### 5.1. Zero-dimensional (reduced-order) models

Compartmental, or ‘0D’, models are computationally inexpensive, rendering them highly efficient for parameter estimation in clinical applications. These models use simplified, 0D compartments to model the mechanical behaviour of respiration [105–111]. Both the number of compartments and their connectivity depend on the application. For each compartment, ordinary differential equations may be used to describe lung deformation, gas exchange, gas transport [106] and parenchymal interdependence [6]. The compartmental nature of these models also means that different anatomical components of the lung (e.g. capillaries, alveolar septal wall, alveolar space, airways, etc.) can be accounted for, leading to anatomically detailed lung models with larger parameter spaces. The interaction between compartments can be also described using additional differential equations [6,112].

**Figure 5.**
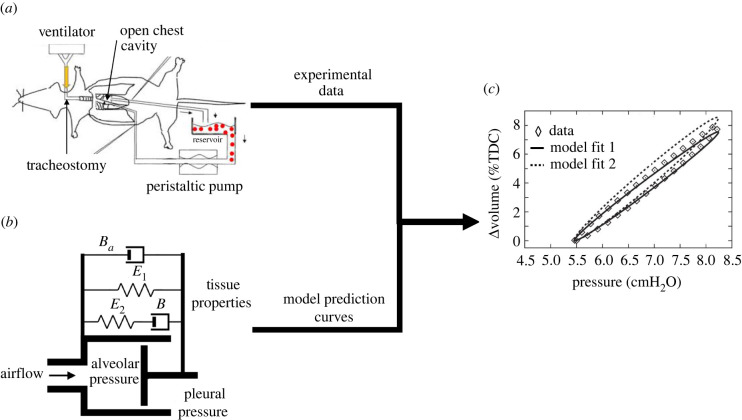
Schematics of the steps in developing a compartmental computational model and estimating lung properties. Images taken from [6,12,112] with permission. (*a*) Invasive P-V measurements, (*b*) compartmental model and (*c*) model fits to experimental data. TDC, total duct capacity.

A common workflow for preclinical compartmental models is shown in figure 5. Parameter estimation depends on the acquisition of lung function measurements, which are typically acquired invasively in murine/rodent models via intubation of the trachea for ventilation (figure 5*a*). Compartmental models typically include parameters for airflow and acinar mechanics, represented by a combination of resistors, springs and dampers (figure 5*c*). These unknown parameters can be quantified by fitting the model prediction for lung function to the experimental measurements (figure 5*c*). Because these models enable the quick estimation of lung function parameters (rather than waiting for periodic scans), they are useful in clinical situations where the patient’s condition is rapidly changing. Given the low computational expense of these models and their ability to work with limited and rapidly changing data, they are commonly employed clinically for the real-time assessment of lung health and adjustment of mechanical ventilation settings in ARDS patients. Also, these models can be coupled with higher-order models (as described in §5.3.3) to capture the multiphysics nature of lung biomechanics while keeping computational costs reasonable [113].

### 5.2. Discrete micromechanical models

**Figure 6.**
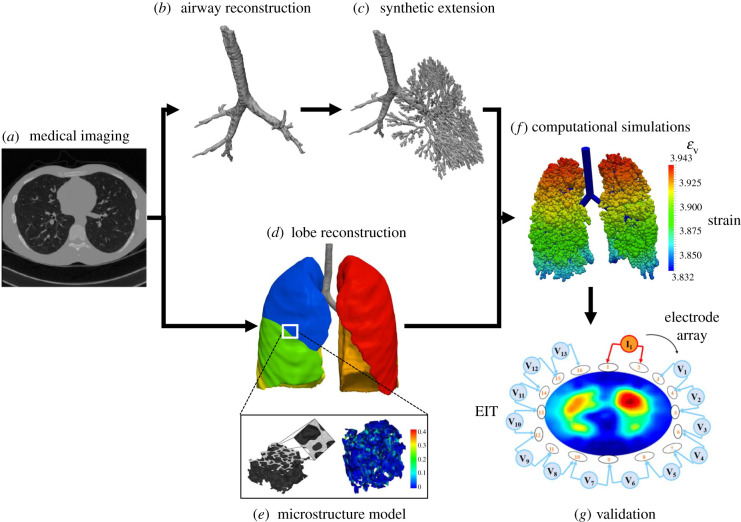
Schematics of the steps in developing an image-based computational model. EIT, electrical impedance tomography; *ε*_v_, volumetric strain. Images taken from [6,7,13,58] with permission.

Microscale models of portions of the lung are instrumental to the creation of accurate and predictive computational models. Discrete micromechanical modelling is a common approach for studying the mechanical behaviour of lung parenchymal tissues at smaller scales using a representative tissue element (RTE). The RTE captures the tissue’s behaviour at a ‘mesoscale’ that contains sufficient information about tissue microstructure (microscale) and is expected to statistically represent the behaviour of larger tissue volumes (macroscale) (figure 6). The behaviour of the RTE can therefore be used to develop material models that represent lung tissue stress–strain response. Two types of discrete mechanical models that will be covered here: two-dimensional (2D) spring models and 3D acinar unit models.

#### 5.2.1. Two-dimensional spring network models

Two-dimensional spring models represent the alveoli as a network of 2D springs, with hexagonal units being the most common approach. Spring models are computationally efficient and can be used to explore the phenomenon of alveolar interdependence. Mead *et al.* [114] modelled the alveoli as a hexagonal network to estimate the distension pressure within individual alveoli and compare it to transpulmonary pressure, demonstrating that the distension pressure is close to transpulmonary pressure in uniformly expanded lungs. Hexagonal models are often made of elastic line elements to simulate naturally observed deformation in the alveoli, with angular springs (or torsion springs) at the nodes to prevent the folding of neighbouring line elements. The use of angular springs at the nodes was introduced in later studies [33,115,116] to simulate alveolar interdependence since the deformation of each hexagonal unit is coupled with deformation in the surrounding hexagonal units. Similar hexagonal alveolar models were developed [33,115,116] to imitate observed deformation patterns, as well as to study the interaction between collagen and proteoglycans [33] and the elastic and hysteresis behaviour of the parenchyma [115].

Cavalcante *et al.* [33] developed a nonlinear hexagonal spring network model to distinguish between the contributions of collagen and elastin. The model also imposed a rotational strain energy function to restrict the free rotation of the springs about the nodes. Experimental studies were performed at different levels of osmolarity to isolate the effect of collagen and elastin, as the mechanical behaviour of proteoglycans is reported to be strongly dependent on osmolarity. Estimating the elastic modulus of the spring representing the alveolar wall, they were able to predict collagen and elastin fibre moduli. Another recent study using spring models was reported by Jawde *et al.* [20] in which the lung parenchyma was modelled as a hexagonal network of springs to investigate the mechanism of alveolar wall deformation. Alveolar walls bend and extend under deformation, and this study was able to separate the contribution of the two mechanisms and compute the relationship between macroscopic tissue strain and microscopic alveolar strain. They were also able to validate the model against both tensile tests of parenchymal tissue strips and mechanical behaviour observed in the literature [33,117].

#### 5.2.2. Three-dimensional acinar unit models

Acinar and alveolar models constitute another important class of micromechanical parenchymal models. Acinar models involve simulating a large number of acinar structures that are inflated using air pressure at the terminal airways [37,40,52,118]. Alveolar models tend to focus on a single alveolus [7,85,119,120] and have been used to study the effect of surface tension on the expansion of the alveoli [7,120]. Acinar models aim to provide a structurally accurate picture of the parenchyma by including numerous acinar units, each of which models an acinus and behaves similarly to a balloon expanding under pressure. Despite significant resemblance to lung acinar structure, these models often do not account for parenchymal interdependence—i.e. the fact that the expansion of an alveolus depends on its surrounding alveolar units. Inter-acinar interactions need to be accounted for to generate a realistic deformation at the parenchymal tissue scale but are often omitted to reduce computational complexity. Additionally, acinar models are computationally expensive since they involve simulating thousands of physical units for an accurate prediction. Several such models reported in the literature are reviewed below.

Weichert *et al.* [120] developed an alveolar model based on a biophysical approach that accounted for the varying surface tension of the liquid film. They modelled a single alveolar sac as a TKD, or a truncated octahedron, and incorporated the cyclic change in surfactant concentration in the liquid film during breathing by including an interfacial energy term on the inner surface of the TKD (alveolar) element. They demonstrated the effect of the surfactant in reducing the surface tension of the liquid film lining, thereby reducing the stiffness of the alveoli. Wall *et al.* [7] used a similar acinar model as an RTE to predict larger-scale parenchymal tissue behaviour. They demonstrated their multiscale approach by simulating the deformation of a heterogeneous parenchymal tissue strip due to inflation (respiration), where the local behaviour was derived from an assembly of acinar units with different properties.

The work by Swan *et al.* [121] investigated the effects of gravity on the topographic distribution of ventilation in the lungs in the upright posture, to address the lack of detailed upright imaging data (since most imaging methods require the patient to be lying supine). They computed the changes in ventilation by including acinar units at the end of the airways expanding isotropically due to airflow, while the flow rate and pressures at the terminal airways were computed assuming a line transmission model. The study found non-uniform acinar volume at FRC, with acini at the apex containing higher volume than acini at the base of the lungs, resulting in higher normalized ventilation at the base compared with the apex. The authors also found that lung compliance plays a more significant role than airway resistance in determining ventilation distribution in healthy patients.

An example of coupling reduced-order models with higher-order models is the work by Roth *et al.* [52], where the authors combined a reduced-order acinar model with a (higher-order) synthetic airway tree model to determine the variation in ventilation caused by atelectasis. The authors used CT scans to determine atelectatic regions. They then closed the airways in those regions and applied appropriate boundary conditions to simulate mechanical ventilation. They reported that the closure of airways in atelectatic regions may prompt over-ventilation of healthy regions under default mechanical ventilation protocols that, in turn, can lead to over-stretching of alveoli walls and further lung injury. The authors validated their simulation results against EIT measurements (figure 6*g*) of the deformation at the fifth intercostal space, supporting their claim that this model can be used to individualize ventilation protocols based on EIT monitoring.

One limitation of acinar models is the absence of inter-connectivity between the acinar units. This limitation was recently addressed by Ma *et al.* [39]. The authors combined the acinar model developed by Fujioka *et al.* [37] with surfactant effects described in Ryans *et al.* [40,41] to develop a half-lung (half of one lung) model with synthetic airways. Each alveolus was modelled as a truncated octahedron, and the model accounted for surfactant transport during the respiration cycle. The interactions between acinar units and flow through the airways were incorporated using reduced order models to reduce the computational demand of the whole model. The resulting model allows for the adjustment of surface tension inside each alveolus (through surfactant concentration), and successfully recapitulated the P-V relationship for the entire lung. As the authors point out, one significant potential application of this model would be to investigate lung injuries where airways are blocked by liquid accumulation leading to regional under-ventilation, with concomitant over-ventilation in healthy lung regions, inducing further lung injury.

Concha *et al.* [119] proposed a method to improve the computational efficiency of lung modelling using a micromechanical parenchymal model consisting of incompressible neo-Hookean structural elements arranged in the shape of a TKD where each TKD represented an alveolar unit. The model predictions were compared against deformations from a finite-element simulation of an RTE reconstructed from micro-CT scans. The TKD-based model was able to reproduce the RTE response for isotropic volumetric expansion, select anisotropic deformations and equibiaxial tension. The primary parameters for capturing the macroscopic behaviour of lung tissues were found to be the porosity and the alveolar wall stiffness. A key advantage of using a structural model (such as TKD) is that it is more numerically stable at higher strains and offers reduced computational cost compared with the finite-element model generated from micro-CT images (discussed below). Concha *et al.* [85] further extended their TKD model to describe the pressure–volume relationship in the lungs during respiration and reported that the computational speed of the TKD model is five orders of magnitude higher than that of its counterpart finite-element model. The authors used their model to perform a parametric study on the sensitivity of parenchymal mechanical behaviour to changes in material properties, simulating the potential effects of diseases such as emphysema (increased compliance) and pulmonary fibrosis (increased stiffness) on lung biomechanics.

#### 5.2.3. Liquid film model

The liquid film present in the alveolar walls is a critical aspect of microscale lung structure. This thin liquid film is instrumental to the lung’s behaviour under both healthy and pathological conditions, as the surface tension can exert large stresses due to the very small curvature radii of the alveoli and acini. The importance of this lining fluid is demonstrated by the dramatic change in PV hysteresis with the removal of the air–liquid interface [106,122]. Functional pulmonary surfactant is critical to the developing lung, and its deficiency is associated with respiratory distress syndrome [123,124] as well as ARDS [125].

Biophysical models of liquid film fluid dynamics under pathological conditions have focused on airway closure resulting from a liquid film instability that creates a meniscus that obstructs airflow [126–128] and can induce a coupled response that collapses compliant airways [129,130]. Surfactant can stabilize this system, and accurate computational models are essential [38,131]. Once an airway or alveolus is collapsed, the motion of the meniscus strongly influences flows and pressures [132,133], and the reopening of airways and alveoli is a very complex fluid–structure interaction [134,135] that can exert enormous stresses on pulmonary epithelial cells. This may induce further damage to the diseased lung [136–139]. Surfactant delivery through aerosols or liquid administration is a highly complex phenomenon that may be important for successful ARDS treatment [140–142].

### 5.3. Continuum models

While discrete alveoli and acini-based models offer insight into the biomechanics of alveoli in respiration, modelling the whole lung with these approaches quickly becomes computationally infeasible due to the large number of acini present and the inter-dependence of alveoli during expansion. An alternative approach to organ-scale lung modelling is to develop a continuum material that can capture the stress–strain behaviour of the lung at the parenchymal tissue scale while also incorporating information about microscopic features such as porosity (figure 6*d*,*e*).

#### 5.3.1. Phenomenological solid models

Phenomenological solid models treat the parenchyma as a non-porous but compressible homogeneous solid (figure 6*d*). While these models do not explicitly account for tissue porosity, tissue compressibility can be appropriately gauged to capture the effects of tissue porosity at the macroscopic level. Phenomenological models were primarily used to study the interaction of the lung with its surrounding tissues such as the diaphragm. Tawhai *et al.* [48] used a homogeneous solid model to determine ventilation differences (measured by tissue density and elastic recoil) due to patient position, arguing that, since lung imaging often requires the patient to lie supine or prone, additional post-processing is needed to translate ventilation results to the case of upright positioning. The parenchymal tissue was modelled using an exponential strain energy function, and the study determined the ventilation difference (tissue density) and tissue recoil pressure as a function of height. However, as the authors note, the assumption of homogeneous parenchyma is likely not valid in the case of lung injury or disease.

Ladjal *et al.* [47] used a similar model to study the effects of lung cancer using four-dimensional (4D)-CT imaging, modelling the lungs and all the interacting elements in the thoracic cavity, including the diaphragm and rib cage, and investigating the interaction between these elements and the lungs using 4D-CT. The ribs were modelled as rigid bodies rotating about their attachment to the spine, while the lungs were modelled as a compressible isotropic hyperelastic material. The authors developed an optimization algorithm to determine the diaphragmatic force using a 4D-CT image-based inverse model. Reported applications included creating a digital duplicate of the lungs that can be used for diagnosis and dose distribution simulation.

#### 5.3.2. Phenomenological poroelastic models

Porosity carries information about the regional ratio of air-to-tissue volume and is an important parameter for characterizing air diffusion throughout the lung tissue. Indeed, the heterogenous distribution of instantaneous porosity is a key determinant of regional alveolar deformation that needs to be accounted for to accurately model the ventilation behaviour. Structurally based poroelastic models can feasibly simulate organ-level behaviour validated by CT scan and volumetric measurements, and can statistically represent smaller scale structural features of the lung. Berger *et al.* [5] modelled the parenchyma as a poroelastic material characterized by a neo-Hookean strain energy function. Normal breathing was simulated using a displacement boundary condition on the surface of the lungs at FRC and TLC, as measured from CT scans. Air was then assumed to completely fill the porous material, simulating lung ventilation. Mechanical properties of the lung parenchyma were obtained from experimental studies [143,144]. This model was used to study the effects of localized airway constriction on lung ventilation by reducing the radii of the terminal airways in a certain region. A large elastic stress was found at the boundary between healthy and affected tissue due to the difference in ventilation (i.e. stress).

Patte *et al.* [145] developed a similar poroelastic model to study lung deformation driven by the gradient between pleural and alveolar pressure. Their study also accounted for interaction with the ribcage as a means to limit deformation. This work was extended by Genet *et al.* [146] to study pulmonary fibrosis, where the fibrotic tissue was segmented using CT scan images and material properties were determined using an inverse model. The authors compared the ventilation behaviour of healthy versus diseased lungs and reported that diseased tissue was stiffer than healthy, in agreement with commonly observed tissue stiffening in pulmonary fibrosis [147].

#### 5.3.3. Image-based poroelastic models

Image-based poroelastic models involve reconstructing microstructurally faithful geometries of alveolar tissues, often obtained using micro-CT and developing finite-element models of using these geometries. The acquired geometry allows for modelling realistic alveolar microstructure, as opposed to the phenomenological poroelastic model that assumes an average size for the pores (alveoli). Unlike an idealized geometry, this approach allows the modelling of diseased tissue to be used to individualize treatment protocols in a clinical setting. However, the primary limitation of a finite-element approach is the computational cost, with the average time for a forward simulation of the cubic specimen (of size 100 μm) being 300 min [85], limiting the specimen size for inverse problems. However, under the condition of statistical homogeneity in healthy lungs, a micro-CT based finite-element model of a parenchymal cube can be used to develop a ‘homogenized’ behaviour described by a continuous energy function [85,119].

Rausch *et al.* [59] developed an imaged-based finite-element model of a cubic rat lung specimen using micro-CT scans. Whole lung samples were kept at a fixed physiological pressure to prevent alveolar collapse. The cubic element (with a dimension of 100 μm) was isolated from the images to generate a finite-element mesh, which was subjected to tensile and shear loading. The authors used the model to determine the relationship between maximum local strain and macroscopic strain experienced by the cube. Interestingly, they found that thin alveolar walls are subjected to strains up to four times larger than the macroscopic strain and that such hot spots were present only in certain septal walls in the cube. These findings imply that alveolar walls may be at risk for overdistension and epithelial cell damage when the macroscopic strains are at safe values.

In a related study, Sarabia-Vallejos *et al.* [11] used micro-CT to create a finite-element model of a section of the lung to estimate alveolar stresses. The authors used rat lungs to investigate the effects of alveolar pressure and porosity on the stress sustained by the alveolar septal wall. Like in the previous study, the whole lung was imaged and two cubic elements (100 μm and 300 μm in size) were isolated to generate finite-element meshes, which were subjected to hydrostatic pressure loading. They found that von Mises stress on the alveolar wall could be 12–27 times the alveolar pressure. The study also found that 2D mechanical analyses tend to overestimate alveolar stresses [148]. However, the authors acknowledged the important limitation of not considering surfactant concentration kinetics in their modelling. The presence of surfactant can indeed significantly modulate the amount of mechanical stress experienced by the alveolar wall, as the surface tension forces induced by surfactant are believed to accommodate a large fraction of alveolar pressure.

### 5.4. Fluid dynamics models of ventilation

Physiological respiration involves a heterogeneous distribution of pressure within the lung, so comprehensive modelling of the lungs will depend on knowledge of this pressure field. In turn, air flow and pressure determine the deformation sustained by the alveoli and can directly contribute to pathologies involving alveolar over-distension. The pressure field is determined by knowing the air flow in the conducting airways. Hence, to understand the ventilation distribution in different regions of the lungs, it is necessary to understand the mechanics of airflow through the airways [149]. Early studies investigating the flow through the airways used a mathematical fractal tree model developed by Weibel [18] and Horsfield *et al.* [15] to study gas mixing, flow distributions, and aerosol deposition [150–152]. More recent advances in generating the airway tree structure were reported by Tawhai *et al.* [153], who obtained the lung and proximal airway morphology from MRI scans and generated the distal airway tree structure ‘synthetically’ using a space filling algorithm and lung morphology (figure 6*c*).

The ability to reconstruct anatomically faithful models is limited by the resolution of the CT scans used; however, since the flow velocity and Reynolds number in distal branches are small, they can be represented by 1D transmission element models [118,154]. Bordas *et al*. [17] used MDCT scans to develop computational fluid dynamics (CFD) models of air flow in central airways to investigate the flow resistance in healthy and asthmatic patients. The study showed a significant increase in airway resistance with asthma, and the estimated resistance from CFD simulations showed a strong correlation with both patients’ forced expiratory volume in one second (FEV1) and the ratio of FEV1 over forced vital capacity (FEV1/FVC).

Interestingly, while it is important to include the airway tree in a lung model to capture ventilation distribution, a study by Ma *et al.* [118] investigated flow in the upper airway (mouth to trachea) using forced oscillation simulations, where the frequency of the input was between 0.156 Hz and 8Hz. This study used proton MRI and CT scans to generate the upper airway and the airway tree up to the sixth generation, respectively. The nasal passage was not considered due to insufficient MRI resolution to capture its complex structure, and so was manually closed off in the model. CFD simulations showed that the upper airways account for 45–70% of total resistance at 0–1 Hz and 70–81% of total lung resistance between 1 and 8 Hz. The trachea did not affect resistance but contributed significantly to the elastance of the lungs.

Overall, CFD models of ventilation can provide significant insights into the mechanisms and flow alterations in pulmonary diseases involving alterations in airway structure such as bronchoconstriction (e.g. asthma), bronchopulmonary displasia, tracheomalasia, COPD, etc. Understanding how these diseases disturb the flow in the airways is essential in modelling and optimizing pulmonary drug delivery techniques such as aerosol transport. Studies by Venegas *et al.* [155] and Donovan *et al.* [156,157] investigated the effect of bronchoconstriction, induced by asthma, on the airflow in the lungs. Venegas *et al*. used PET scans to demonstrate that bronchoconstriction leads to regions of poor ventilation where the injected tracer is unable to be washed out of the lungs. This condition was referred to as bimodal ventilation, implying that there are two levels of ventilation in the lungs. Venegas’s study further used a simplified airway structure model to mimic similar effects when parts of the airway tree were blocked. Donovan *et al.* [156] developed a realistic airway tree to simulate the airway flow behaviour in asthma through randomly generated constrictions, and they found that a greater number of bronchioles are underventilated compared to the control lungs. Their study confirmed a bimodal ventilation distribution that has also been observed experimentally in rats [158].

## 6. Future directions

Computational lung biomechanics research holds immense potential for improving our understanding of the structure–function relationship in the lung. The lung’s complex and hierarchical anatomy is a challenge in developing physiologically realistic lung models, demanding multiscale approaches that capture the airway tree, alveolar network structure and local phenomena at the alveolar scale (such as surfactant activity), while analysis is further complicated by the presence of an air–liquid interface with dynamic surface tension. Below, we discuss several ways in which we believe computational lung modelling can improve our understanding of both basic science and translational aspects of lung biomechanics.

### 6.1. Subject-specific lung modelling advanced by machine learning

Despite the several studies on image-based lung modelling reviewed in this paper, there remains a need for subject-specific lung models that can incorporate commonly available imaging and functional data. Personalized computational models of the lung will improve the diagnosis and treatment of various respiratory illnesses and facilitate a more comprehensive understanding of lung function in health and disease. Current image-based computational studies which use medical imaging are computationally expensive [5,52,145,153], with inverse modelling simulations requiring days/weeks to complete. For critical care applications, models need to work with limited data (e.g. pressure–volume measurements and a potential CT or EIT scan) and be able to generate predictions in minutes, as a patient’s condition may rapidly evolve. Compartmental models that have a limited parameter space and require limited functional data may meet this need; however, they may fall short in the case of lung diseases with strong spatial heterogeneity, requiring the incorporation of image-based morphological and microstructural information for reliable prediction. There is thus a need to combine the computational advantages of compartmental models with the multiscale models’ ability to account for high-fidelity properties to create computational models which balance personalization, accuracy and predictive speed. Nonetheless, image-based high-fidelity models still serve as a promising platform for identifying, isolating and studying the mechanisms of disease progression.

Recently, machine learning (ML) techniques have proven helpful in enhancing the translation of computational lung modelling to the clinic [159,160]. Although ML often is directly applied in lung imaging to study clinical aspects of lung function such as ventilator parameter optimization [161,162], ML toolsets can also be used to facilitate several steps in common image-based modelling pipelines, that would otherwise require time-intensive processes. These steps include segmentation of the lungs and airways [163–165], isolation and segmentation of diseased regions such as inflammation, obtaining strains and displacements from image registration, and so on. The use of ML to automate these, otherwise tedious, steps would allow these processes to be performed in timelines suitable for timely patient-specific treatment decision making in clinical applications. In addition, image-based computational models, requiring CFD or finite-element simulations, could entirely be replaced by trained ML models [166]. Altogether, the ML models hold a strong promise to significantly augment individualized diagnosis, prognosis, and therapeutics in pulmonary diseases.

### 6.2. Multiphysics, multiscale models of the lung

Multiphysics and multiscale modelling of the lung remains an area of active research, showing promise for full integration of different respiratory mechanisms in the lung. Respiration is a complex process that takes place across multiple length scales and involves tissue deformation, air flow, gas exchange in the alveoli and blood flow through the capillaries. These phenomena are intertwined, demanding a multiphysics modelling approach to accurately capture their interaction and contribution to lung function. A multiscale approach to modelling the structure–function relationship in the lung is also indispensable. Lung deformation at the tissue level is driven by the recruitment and deformation of the alveoli [89]. During inflation, the alveoli go through recruitment (if already collapsed), septal unfolding, changes in shape, and then septal stretching—with recruitment being unlikely during regular respiration. Here, the shape change is dominated by the effect of surfactant, while the stretching phase is dominated by the elastic properties of the ECM. A multiscale model is needed to connect these complex alveolar micromechanics to organ-level function [167]. Such a model will address the need to predict changes in the organ-level function based on alterations in alveolar behaviour, and vice versa. In addition, alveolar micromechanics itself is largely influenced by the make-up of the septal wall fibrous network involving collagen, elastin, and proteoglycans. Further down-scaling of micromechanical models can then provide valuable insights into the contribution of each fibre group to the parenchymal mechanics at larger length scales.

Another related area of research is the development of physiological material models for tissue-level behaviour to be incorporated into inverse models. The hysteresis observed in the P-V loop of the lungs is largely due to the surface tension of the liquid film [106] in the alveoli and the regulation of its surfactant concentration. The effect of varying surfactant concentration has been studied using acinar models [120] but has not been incorporated directly into the material properties of continuum models. Continuum poroelastic models of parenchymal tissue that incorporate the effects of surface tension and viscoelasticity will allow for improved simulation of respiratory biophysics in the whole lung.

### 6.3. Growth and remodelling

Developmental and pathological growth and remodelling (G&R) studies could provide valuable insights into the progression of different lung diseases. G&R often refer to changes to one or more of the tissue properties in response to lung disease or injury—including mass, volume, material properties and architecture. G&R models have been developed to investigate biomechanical alterations of different soft tissues in disease and to identify the mechanisms driving these alterations [168,169]. Humphrey [170] reviewed constrained mixture models that have been applied to investigate the G&R of soft tissue with a focus on vascular tissues. Lung G&R models could adopt and build on the G&R frameworks developed in the vascular field to model and understand lung remodelling processes in pulmonary diseases. For instance, the study by Hill *et al.* [171] offers a modelling foundation to study inflammation-driven airway remodelling in asthma. Such studies will improve our understanding of underlying causes/drivers of remodelling events, potentially enabling the prediction of chronic remodelling events, which could in turn facilitate individualized interventions.

## Data Availability

This article has no additional data.
